# The Influence of Filler Particles on the Mechanical Properties of Maxillofacial Prosthetic Silicone Elastomers: A Systematic Review and Meta-Analysis

**DOI:** 10.3390/polym12071536

**Published:** 2020-07-12

**Authors:** Aparna Barman, Farah Rashid, Taseef Hasan Farook, Nafij Bin Jamayet, James Dudley, Mohd Firdaus Bin Yhaya, Mohammad Khursheed Alam

**Affiliations:** 1 Maxillofacial Prosthetic Service, Prosthodontic Unit, School of Dental Sciences, Health Campus, Universiti Sains Malaysia, Kubang Kerian, Kota Bharu 16150, Kelantan, Malaysia; aparnabarman256@gmail.com (A.B.); qazifarahrashid@gmail.com (F.R.); taseef@live.co.uk (T.H.F.); 2 Adelaide Dental School, The University of Adelaide, Adelaide 5005, South Australia, Australia; james.dudley@adelaide.edu.au; 3 Advisor for Biomaterial and 3D Visualization Lab, School of Dental Sciences, Health Campus, Universiti Sains Malaysia, Kubang Kerian, Kota Bharu 16150, Kelantan, Malaysia; firdausya@usm.my; 4 College of Dentistry, Jouf University, Sakaka 72345, Saudi Arabia; dralam@gmail.com

**Keywords:** filler particle, hardness, maxillofacial silicone, tear strength, tensile strength

## Abstract

Although numerous studies have demonstrated the benefits of incorporating filler particles into maxillofacial silicone elastomer (MFPSE), a review of the types, concentrations and effectiveness of the particles themselves was lacking. The purpose of this systematic review and meta-analysis was to review the effect of different types of filler particles on the mechanical properties of MFPSE. The properties in question were (1) tensile strength, (2) tear strength, (3) hardness, and (4) elongation at break. The findings of this study can assist operators, technicians and clinicians in making relevant decisions regarding which type of fillers to incorporate based on their needs. The systematic review was performed according to Preferred Reporting Items for Systematic Reviews and Meta-Analyses (PRISMA) guidelines. A total of 26 original articles from 1970 to 2019 were selected from the databases, based on predefined eligibility criteria by two reviewers. The meta-analyses of nine papers were carried out by extracting data from the systematic review based on scoring criteria and processed using Cochrane Review Manager 5.3. Overall, there were significant differences favoring filler particles when incorporated into MFPSE. Nano fillers (69.23% of all studies) demonstrated superior comparative outcomes for tensile strength (*P* < 0.0001), tear strength (*P* < 0.00001), hardness (*P* < 0.00001) and elongation at break (*P* < 0.00001) when compared to micro fillers (30.76% of all studies). Micro fillers demonstrated inconsistent outcomes in mechanical properties, and meta-analysis of elongation at break argued against (*P* < 0.01) their use. Current findings suggest that 1.5% ZrSiO_4_, 3% SiO_2_, 1.5% Y_2_O_3_, 2–6% TiO_2_, 2–2.5% ZnO, 2–2.5% CeO_2_, 0.5% TiSiO_4_ and 1% Ag-Zn Zeolite can be used to reinforce MFPSE, and help the materials better withstand mechanical degradation.

## 1. Introduction

Maxillofacial prosthetic silicone elastomers (MFPSE) are considered an appropriate prosthetic material for skin-like replacement, but have been plagued by issues such as mechanical degradation, color instability and short life span [1]. The issues are usually caused by environmental factors resulting in the need for the frequent prosthesis changes. The most clinically significant properties of MFPSEs, along with their recommended values, have been demonstrated in the previous literature as: tensile strength 6.90–13.8 megapascal (MPa); tear strength 5.25–17.51 N/mm; hardness 25–35 shore A; and elongation at break of 400–800% [2,3,4,5]. But in reality, a pure mix of MFPSE alone seldom yields the desired values, and undergoes material degradation and weathering as a consequence after using for a certain period of time [3,4,6]. To combat the said issue, various organic and inorganic filler particles of different types and sizes have been incorporated into MFPSE. These filler particles reinforce MFPSE and allow the material to better-withstand normal function and weathering [7].

Although numerous studies have demonstrated the benefits of incorporating filler particles [7,8,9], a review of the types, concentrations and effectiveness of MFPSE fillers is lacking. The purpose of this systematic review and meta-analysis was to review the effects of different types of filler particles on the mechanical properties of different maxillofacial MFPSE’s.

## 2. Materials and Methods

### 2.1. Literature Search Strategy

This study was conducted according to the Prisma (Preferred Reporting Items for Systematic Reviews and Meta-Analyses) 2009 checklist [10], and performed in two parts:

1. A systematic review of all studies that reported on the mechanical properties of filler particles and MFPSEs. (tensile strength, tear strength, hardness, elongation at break).
2. A meta-analysis of studies that compared the mechanical properties between MFPSE incorporating filler particles and MFPSE without filler particles. These studies were extracted from the systematic review according to scoring criteria.

A literature search of publications from 1970 to December 2019 was conducted using the Scopus and Web of science databases. Between 1 November 2019 and 10 January 2020, two reviewers searched in parallel and reviewed the search results independently then collaborated. The search strategy enabled an exhaustive search of the available published literature.

#### 2.1.1. Search of Study Design

The following search words were used:

“Maxillofac* + silicon* + elastomer + prosthe*”, “Mechanical + properties + silicon* + maxillofac*”, “Prosthe*+ silicon* + facial + oxide”, “Maxillofac*+ titanium + oxide + prosthe*”, “elastomer + nano particles + maxillofac* + properties”, “maxillofac* + aging + hardness + silicon*”, “Maxillofac* + nano + oxide + silicon*+ prosthe*”, “Facial + prosthe* + oxide + strength”, “Mechanical + oxide + silicon* + maxillofac*”, “Elastomer + aging + mechanical +hardness”, “aging + prosthe* + filler + strength”, “maxill* + prosthe* + silicon* +silica”, “Weather* + prosthe* + filler + strength”, “Silicon* + TiO_2_ Prosthe*”, “Prosthe* + Silicon* + ZnO”, “Filler + strength + maxillofac* + silicon*”, “silicon* + micro* + maxillofac* + propert*”, “silicon* + poly* + maxillofac* + propert*”, “silicon* + fib* + maxillofac*+ propert*”, and “silicon* + PMMA* + maxillofac*+ propert*”

#### 2.1.2. Inclusion and Exclusion

The inclusion criteria were:

- Comparative studies on MFPSE that included filler particles;
- Studies where MFPSE was not subjected to chemical stimulus influence (for example, detergent and alkaline solution) prior to mechanical property testing;
- Articles describing changes in tensile strength, tear strength and/or hardness and elongation at break of MFPSE.

The exclusion criteria were:

- Articles that compared MFPSE without any filler involvement;
- Studies where MFPSE specimens were incorporated with pigments prior to testing filler properties;
- Articles in other languages without accompanying English translations;
- Review articles and case reports on silicone elastomer not involving maxillofacial region;
- Unpublished literature.

### 2.2. Data Collection

After obtaining all search results based on title screening, duplicates were removed using Endnote (version X8.2, Clarivate Analytics, Toronto, ON, Canada) followed by abstract screening then full paper reviews. The reviewers consulted after reading each paper to maintain consensus. The articles for complete review were assessed by both reviewers and the reason(s) for exclusion were documented. A Kappa concordance analysis was conducted to determine the inter-rater reliability agreement.

#### 2.2.1. Search Items (Data to Be Extracted from Articles)

The following search items were used: type of silicone used, number of specimens, presence or absence of filler particles, types of filler particles, concentration of filler particle (in percentage), type of weathering, tensile strength properties, tear strength properties and hardness and elongation at break.

#### 2.2.2. Summary Measures

The metric units used as standard for this study were megapascals (MPa) for tensile strength, newton millimeters (N/mm) for tear strength, (shore A) for hardness and percentage (%) for elongation at break. Metrics were converted to the standard units where appropriate to aid in the correlation of values.

### 2.3. Meta-Analysis Scoring Criteria

The conditions of meta-analysis were formulated according to the Newcastle–Ottowa scale [11], modified for material-based case-control reports. The methods of presenting results for quality and exclusion were based on previous similar work by Scurria et al. [12] (Table 1). Articles that satisfied all requirements and had a score of three were considered for meta-analysis.

### 2.4. Synthesis of Results

The results of the meta-analysis were synthesized using the statistical method of inverse variance in random effects with a 95% confidence interval. Cochrane Review manager 5.3 (Cochrane Library, London, UK) was used to tabulate the results according to the scoring criteria. Cochrane Review Manager was used to derive statistical heterogenicity, *P* values, Z values and subsequent subgroup analyses with the forest plots.

## 3. Results

**Figure 1:**
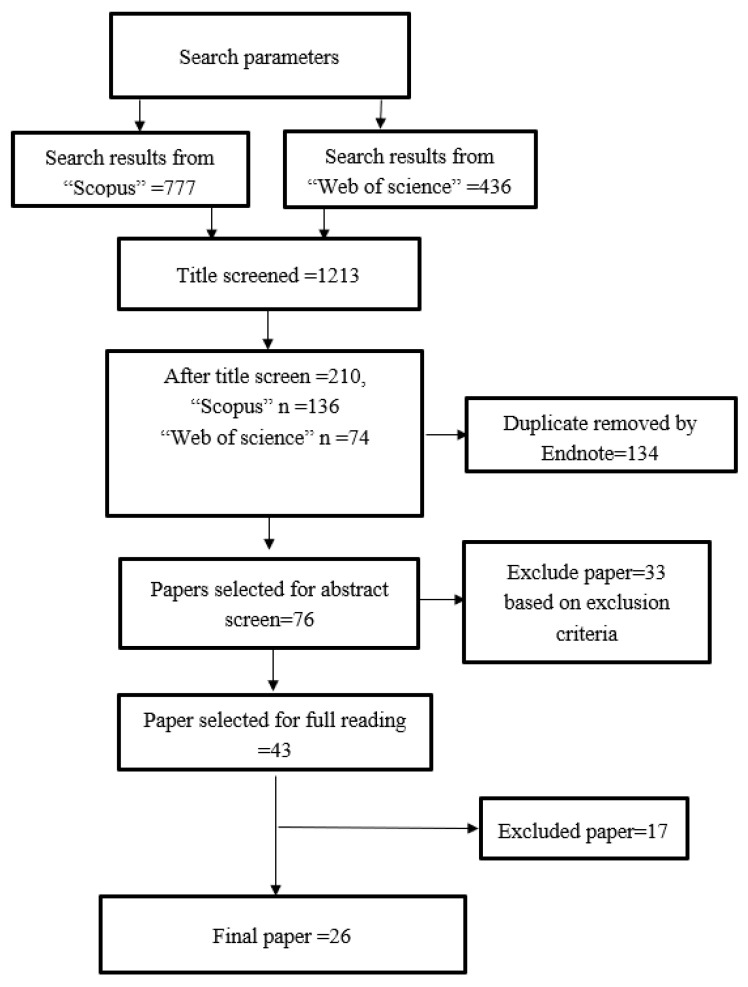
Prisma flow-chart on article screening.

A total of 1213 studies were initially sourced from the databases. Following title and abstract screening, 43 publications were selected for full reading, of which 26 papers were selected and 17 papers were excluded (Figure 1). The Kappa concordance inter-rater reliability agreement (K score) was 0.85, indicating almost perfect agreement.

The mechanical property changes, associated with various types of filler particles for the 26 included publications, are listed in Table S1. Among the 26 studies, quality scoring according to predefined criteria yielded nine studies eligible for meta-analysis. The scoring and reasons for exclusion of the remaining studies are presented in Table 2.

### 3.1. Synthesis of Meta-Analysis Results

Meta-analysis was conducted for nine studies, as highlighted in the bold text within Table S1. The studies evaluated the changes in tensile strength, tear strength, hardness and elongation at break (seven of the nine studies) of MFPSE incorporated with filler particles.

#### 3.1.1. Tensile Strength

MFPSE without filler particle incorporation was used as the control group and filler-induced MFPSE represented the case group (Table 3). From the varying results it can be found that filler incorporation improved the tensile strength of MFPSE significantly (*P* = 0.002). While the overall forest plot favors the case group, some studies showed inclination towards the control. At a confidence interval (CI) of 95%, I^2^ for tensile strength was 85% with high heterogenicity, Chi^2^ = 116.50, Degree of freedom (df) = 17 (*P* < 0.00001), and overall effect size was Z = 3.09.

Subgroup analysis: The filler-incorporated forest plot showed heterogeneity (Tau^2^ = 1.31; Chi^2^ = 43.43; df = 8 (*P* < 0.00001); I^2^ = 82%) and the test for overall effect [Z = 4.41(*P* < 0.0001)]. The micro filler-incorporated forest plot showed heterogeneity [Tau^2^ = 0.71; Chi^2^ = 33.08; df = 8(*P* < 0.0001); I^2^ = 76%] and the test for overall effect [Z = 0.33 (*P* = 0.74)]. In exploring the subgroup for tensile strength (Table 4) there was a highly significant difference (*P* < 0.00001) when nano filler was added to the material, however there was no significant difference when micro fillers were added (*P* = 0.74).

#### 3.1.2. Tear Strength

MFPSE without filler particle incorporation was used as the control group, and filler-induced MFPSE represented the case group (Table 5). Despite the variety of findings, it was found that incorporating filler significantly improved the tear strength of silicone elastomer (*P* < 0.00001) (Table 5). While the overall forest plot favored the case group, some studies showed an inclination towards the control. At a confidence interval (CI) of 95%, I^2^ for tear strength was 92% with high heterogeneity, Chi^2^ = 208.67, df = 17 (*P* < 0.00001) and there was an overall effect size of Z = 5.23.

Subgroup analysis: The nano filler-incorporated forest plot showed heterogeneity (Tau^2^ = 4.72; Chi^2^ = 101.76; df = 8 (*P* < 0.00001); I^2^ = 92%) and the test for overall effect [Z = 4.53 (*P* < 0.00001)]. The micro filler-incorporated forest plot showed heterogeneity [Tau^2^ = 3.84; Chi^2^ = 84.10; df = 8 (*P* < 0.00001); I^2^ = 90%] and the test result for overall effect [Z = 2.77 (*P* = 0.006)]. In exploring the subgroup for tear strength, there was a significant difference observed when both nano and micro filler particles were added to the material; however, nano fillers showed greater significance (*P* < 0.00001) than micro filler particles (*P* = 0.006) (Table 6).

#### 3.1.3. Hardness

MFPSE without filler particle incorporation was used as the control group, and filler-induced MFPSE represented the case group (Table 7). From the variety of results, it was found that the incorporation of filler significantly improved the hardness of the silicone elastomer (*P* < 0.00001). While the overall forest plot favored the case group, some studies showed an inclination towards the control. At a confidence interval (CI) of 95%, I^2^ for hardness was 87% with high heterogeneity, Chi^2^ = 128.24; df = 17 (*P* < 0.00001) and the overall effect size was Z = 6.46.

Subgroup analysis: The nano filler-incorporated forest plot showed heterogeneity [Tau^2^ = 4.11; Chi^2^ = 86.12; df = 8 (*P* < 0.00001); I^2^ = 91%] and the test for overall effect [Z = 4.64 (*P* < 0.00001)]. The micro filler-incorporated forest plot showed heterogeneity [Tau^2^ = 1.24; Chi^2^ = 38.35; df = 8 (*P* < 0.00001); I^2^ = 79%] and the test result for overall effect [Z = 4.61 (*P* < 0.00001)]. In exploring the subgroup for hardness, there was a significant difference observed when both nano (*P* < 0.00001) and micro (*P* < 0.00001) particles were added to the material, thus favoring the case groups (Table 8).

#### 3.1.4. Elongation at Break

Of the papers included for the meta-analysis, seven articles explored elongation break in the same experimental samples as those used for the aforementioned properties, and therefore these were included. Table 9 shows the forest plot with evidence that filler particle incorporation significantly (*P* = 0.01) increased elongation at break. At a confidence interval (CI) of 95%, the I^2^ for elongation at break was 96% with high heterogenicity, Chi^2^ = 282.81, df = 12 (*P* < 0.00001), and overall effect size was Z = 2.57.

Subgroup analysis: In exploring the subgroup for elongation break (Table 10) there was a highly significant difference (*P* < 0.00001) favoring the addition of nano filler to the material (Chi^2^ = 183.03, Z = 4.82), however significant differences were found for the studies demonstrating against the incorporation of micro fillers (*P* = 0.01, Chi^2^ =38.76 and Z = 2.56).

## 4. Discussion

There have been limited clinical trials performed to test adhesives and bonding [13], and few clinical trials have assessed the mechanical properties of MFPSE [14,15], meaning clinical decisions regarding MFPSE need to be largely based on, and performed with referenced to, in vitro studies.

### 4.1. Nano Particles and Their Effect on Mechanical Properties

The benefits of incorporating fillers [16] into MFPSE has been demonstrated, where color pigments have influenced the material’s mechanical properties. The study of Nobegra et al. [16] showed that hardness decreased by 0.34 shore A after incorporating oil paint into filler-containing MFPSE. The present study analyzed non-pigmented MFPSE specimens made under the same experimental conditions, to allow for comparison of the mechanical property changes induced by the filler particles alone.

The meta-analysis component of the current study indicated that the addition of fillers significantly benefitted the case group with regards to tensile strength, tear strength, hardness and elongation at break; however, after subgroup analyses, nano particles produced more favorable results for all four properties. Titanium dioxide (TiO_2_) is a commonly used filler, and can be safely applied up to a concentration of 25% in cosmetics and silicones [8]. Nano sized silica/silicone dioxide (SiO_2_), titanium dioxide (TiO_2_), zinc oxide (ZnO) and cerium oxide (CeO_2_) are good nano particles, but improvements in the material properties are concentration-specific, and not solely dependent on the type of nano oxide chosen [17].

It has been demonstrated that the addition of 2–2.5% ZnO and TiO_2_ significantly increased tensile, tear strength and elongation at break. In the same study, although the addition of 3% CeO_2_ produced highly significant changes in hardness (3.9 shore A), it simultaneously decreased tensile (−0.6 MPa), tear strength (−0.9 N/mm) and elongation at break (−38%). Therefore, the filler was suggested as only beneficial in concentrations lower than 3%. In a similar study, it was found that the addition of TiO_2_ reduced hardness and it was recommended not to exceed a concentration of 2.5% by weight [15]. Increased hardness makes MFPSE brittle, and therefore is not desirable for clinical use. Nobegra’s study [16] of ZnO demonstrated the same issue with hardness (3.36 shore A) at a 2% concentration. Studies also suggest that an addition of 1% BaSO_4_ to the mix can significantly (1.01 N/mm) improve tear strength. This can be attributed to the high polymerization stability of BaSO_4_ with silicone [5].

Wang et al. [18] and Shakir et al. [8] both experimented with different concentrations of TiO_2_. Wang found significant improvements in tear strength (2.37 N/mm) and elongation at break (51.05%) at 2% inclusion, and improved tensile strength and hardness at 6%. Shakir et al. [8] reported highly significant improvements in tear (4.06 N/mm), tensile strength, hardness and elongation at break at 0.2–0.25%. This may indicate that incorporating TiO_2_ may not be necessary at higher concentrations, as lower concentrations produce significant material property improvements.

Silica (SiO_2_) has been shown to produce significant improvements in both tensile strength and hardness when incorporated at 15% by weight [19]. Zayed et al. found significant improvement at 3% by weight for tensile strength (0.84 MPa), tear strength (26.58 N/mm) and hardness (1.88 shore A). However, Zayed demonstrated that elongation at break had peak results at the 1.5% concentration (164.8%), with subsequent decline at 2–3% [20]. Tukmachi et al. [21] showed significant improvements in tensile (2.11 MPa), tear strength (10.9 N/mm) and elongation at break (278.8%) at a 5% concentration, and hardness was increased by 8.36 Shore A at a 6% concentration of SiO_2_. Cevik et al.’s [17] study suggested that the addition of TiO_2_ to 10% silica yielded the best results (approx. 10.5 Shore A improvement) after weathering. The elongation at break improved in the silica group, and had the lowest values with TiO_2_.

Titanium silicate (TiSiO_4_) at 0.5% displayed insignificant changes to tensile strength (1.00 MPa) and tear strength (4.63 N/mm), and a 1% improvement in hardness (4.00 shore A) [9]. An alternative to TiSiO_4_ could be water-insoluble zirconium silicate (ZrSiO_4_), which produced significant improvements in all four properties (2.84 MPa, 2.65 N/mm, 3.93 shore A and 653.47%) when added at a concentration of 1.5% by weight [6].

Apart from the commonly used nano particles, yttrium oxide (Y_2_O_3_) as a filler at 1.5% concentration can improve tear strength (4.05 N/mm), hardness (5.05 shore A) and elongation at break (58.1%), but made no significant changes to tensile strength (0.76 MPa) [22]. While yttrium particles have antioxidant property advantages [23], they are less effective at improving mechanical properties when acting alone, and therefore should be used in combinations with other nano particles to obtain optimal results.

### 4.2. Micro Particles and Their Effect on Mechanical Properties

The findings from the meta-analysis suggest that micro particles had no significant effect on improving tensile strength and elongation at break when compared to nano particles. Although surpassed by nano fillers in almost every aspect, micro particles produced some amount of improvement in tear and hardness.

Contrary to our overall meta-analysis findings, Liu et al. [24] found Expancel (Akzo Nobel, Sweden), which are spherical, light-weight, hollow microsphere particles used as a damping and insulating material in the drug delivery system, to have the opposite effect. At a 5% concentration, Expancel increased tensile strength (approx. 0.15 MPa) and elongation at break (330%), while it substantially decreased tear resistance (approximately −7.1 N/mm) and hardness (approximately −4 shore A) [24]. Despite the decrease in hardness at the 5% concentration, when the 30% concentration was, given the material’s hardness increased [24]. A further study added 10% SiO_2_ to the 5% Expancel, which once again improved tensile strength (approximately 0.65 MPa) but worsened tear resistance, due to the microcracks forming between the filler and matrix [25]. Hardness only underwent noteworthy improvement (approximately 4.35 shore A) when 15% SiO_2_ was used instead of 10% [25].

Polyamide (Nylon-6)/tulle micro particles, which are widely used in film industries, have also found their way into silicone elastomers [26,27], with their advantageous property being their ability to resist tear without any deformation. Although Gunay et al. [27] did not mention the exact concentration of tulle used, they found a significant increase in tensile (0.597 MPa) and tear strength (16.44 N/mm), but significant decrease in elongation at break (−74%), after tulle incorporation. Alsamaraay et al. [26] used 1% Nylon-6 by weight, and found consistent insignificant changes (−0.24 MPa) for tensile strength over 200 h, as well as for tear strength (−0.87 N/mm) and elongation at break (−67.17%), while improvements in hardness remained significant (2.37 shore A).

Polyester, commonly used for reinforcement coatings, has been incorporated into silicones [28,29] because of its great toughness and flexibility, as well as high weather durability, strength and heat resistance properties [30,31]. Haider et al. [28] used polyester powder at 1%, 3% and 5% concentrations, with significant tensile and tear resistance changes observed at 1%. As seen before, higher concentrations (5%) of the powder were required to obtain significant changes in hardness. Jebur et al. [29] used 2-mm long polyester fibers at 0.25% and 0.5% concentration by weight, and found some improvement for tensile strength (0.730 MPa), hardness (4.168 shore A) and elongation at break (7.22%), but non-significant changes for tear. The highly variable outcomes in properties, from a clinician’s perspective, do not justify the comparatively higher cost and difficulty in manipulation of polyester and polyamides.

Polymethyl methacrylate (PMMA), used for making acrylic dentures and teeth, have also been used as filler particles for MFPSE due to the fact that their incorporation at 5–20% improves the rigidity of silicone [32]. When this theory was put to the test, the tensile strength and hardness improved by approximately 2.4 MPa and 3.5 Shore A, respectively. While 10% PMMA showed promising improvements, all of the material properties decreased when concentrations were increased beyond 10%, but incorporation of PMMA at 5–20% concentration decreased the elongation break values (approximately -110%).

Chitosan powder (Cheng Du Micxy Chemicai., China) is another anti-candidal biomedical micro particle [33] that was incorporated into MFPSE by Al-Judy [34]. At a 3.5% concentration of Chitosan, tear strength improved by 3.18 N/mm, but reduced the tensile strength by 1.23 MPa. Chitosan micro particles demonstrated contradictory results when compared to Liu et al.’s [24,25] study of Expancel micro particles, which reduced tear strength by −7.1 N/mm and marginally improved tensile strength by 0.15 MPa.

Not all micro fillers are controversial in MFPSE. Silver–zinc (Ag-Zn) Zeolite, a commonly used antimicrobial micro particle in catheters and dental restorative materials, demonstrated promising results when mixed with silicone elastomer. At a 1% concentration, the zeolite powder significantly improved tensile strength by 0.45 MPa and tear strength by 7.76 N/mm, while at a 1.5% concentration it improved hardness by 1.49 shore A, but decreased the elongation at break property by 0.5–1.5% [35].

### 4.3. Natural Fillers and Their Effect on Mechanical Properties

All the aforementioned fillers (both micro and nano sized) were chemical- or mineral-based. In recent years, ‘natural’ filler particles have been introduced to reinforce MFPSE [36,37]. The studies used pomegranate peels powder (PPP), seed powder of dates Ajwa (SPDA) at 0.2–0.4% by weight, and ultra-high molecular weight polyethylene (UHMWPE) at 0.5–1% by weight. These materials have preventive and therapeutic effects, but due to their questionable mechanical properties, the natural fillers (0.2–0.5%) were blended with PMMA (5% by weight) before being applied to silicone [36,37].

At a given instance, without re-evaluation after weathering, PPP produced the best results at a concentration of 0.2% (7.888 MPa and 31.1 N/mmfor tensile and tear strengths, respectively), while SPDA produced the best results for all three properties at 0.3% (7.3 MPa, 30.6 N/mm and 30.00 shore A for tensile strength, tear strength and hardness, respectively) [37]. UHMWPE produced the best results at a 0.5% concentration for tensile strength (10.4 MPa) and tear strength (48.00 N/mm), and at 1% concentration for hardness (37.00 shore A) [36]. While PPP has a better tensile strength at lower concentrations, SPDA is more consistent across all the properties at the same concentration.

### 4.4. Synergistic Effects, Laboratory Conditions and Influence of Weather on Filler Properties

Some authors also experimented with different filler combinations to explore their synergistic effects. Aziz et al. [4] concluded in their study that a mix of polydimethylsiloxane (PDMS), 20% silica and 0.28% cross linker significantly improved tear strength (16.62 N/mm). When PDMS concentrations were controlled, the authors found significant benefits at the 20% concentration for tear strength (5.77 N/mm), and at the 30% concentration for tensile strength (1.201 MPa). However, increasing the concentrations of PDMS also decreased elongation at break. Salih et Al [36] found that a combination of 5% PMMA, 0.2% PPP and 0.5–1% UHMWPE produced better mechanical properties.

The majority of the 26 studies tested their samples at standard dry laboratory temperature (23 ± 2 °C) [8,18,20,35]. Some studies tested their samples at room temperature (25–30 °C) [17,24], while one study [32] relied on wet laboratory conditions (distilled water storage) for their samples. Additionally, all the papers within this systematic review preferred artificial weathering to ensure control over weather variations. These studies, while not weathered in geographically varying weathers and seasons, were designed to mimic the weathering process. It is important to note that none of the included papers in the current study explored the effect of weathering on clinically relevant mechanical properties. Thus, the evidence for the fillers’ benefit on weathering as of now is inconclusive, and should be the subject of future discussions. Further research should focus on assessing the chemical changes of MFPSE when incorporating filler particles, and furthermore, clinical trials should be performed to observe the patient-based outcomes. The core mechanical properties of MFPSE vary amongst types and manufacturers, and therefore should also be the subject of a separate research.

### 4.5. Limitations

Upon searching the databases, the authors found no clinical trials that evaluated the mechanical properties of MFPSE in vivo, and hence this meta-analysis was performed using in vitro case-control studies. There were some studies that inadequately described their case and/or control groups, and full data could not be obtained by personal communications. Those studies were excluded from the meta-analysis. Some studies did not clearly mention the mean standard deviation [9,21,34], and so this had to be calculated from graphical representation, which was then scaled.

## 5. Conclusions

Nano fillers were shown to improve the mechanical properties of MFPSE more so than micro fillers. The meta-analysis suggested 1.5% ZrSiO_4_, 3% SiO_2_, 1.5% Y_2_O_3,_ 2–6% TiO_2_, 2–2.5% ZnO, 2–2.5% CeO_2_, 0.5% TiSiO_4_ and 1% Ag-Zn Zeolite can be used to reinforce MFPSE, and help the materials better withstand mechanical degradation.

## Figures and Tables

**Table 1:** Scoring criteria on paper eligibility for meta-analysis.

Score	Criteria
0	Articles do not meet the Population, Intervention, Control and Outcome (PICO) criteria for case definition.
1	Inadequate definition of control: articles do not mention the total sample size and does not give a complete description of control group or sample count.
2	Inadequate comparison of case and control groups on the basis of mechanical property analysis: Inadequate description of mean and standard deviation. Articles with undefined concentration of filler particles. Articles in which tensile strength, tear strength and hardness property were not seen together (i.e., in the same experimental condition).
3	Articles that satisfy case and control definition with adequate comparison of tensile strength, tear strength and hardness property analysis. Articles may or may not include elongation analyses

**Table 2:** Papers excluded from meta-analysis according to scoring criteria, with reasons.

Year	Author	Score	Reason of Rejection
2019	Salih et al.	1	Incomplete sample description
2019	Salih et al.	1	Incomplete sample description
2018	Pınar Çevik	2	Does not include tensile strength property
2018	Al-Hakam J Ibrahim, Hikmat Jameel Al-Judy	2	Does not include hardness property
2018	Salih et al.	1	Incomplete sample description
2017	Cevik and Eraslan,	2	Undefined concentration of filler particles
2017	Tukmachi et al.	2	Inadequate description of mean and standard deviation.
2016	I. Kalamarz	1	Incomplete sample description
2016	Nobegra et al.	2	Did not analyze tensile strength property
2015	Liu et al.	1	Inadequate definition of control
2014	Wang et al.	1	Incomplete sample description
2013	Liu et al.	1	Incomplete sample description
2008	Han et al.	1	Did not give a complete description of control group or sample count
2008	Gunay et al.	2	Undefined concentration of filler particle
2002	Tariq Aziz et al.	0	Does not meet the criteria for meta-analysis, does not have proper control group for comparison and all the groups have filler.
1998	Andreopoulos et al.	1	Incomplete sample description
1994	Andreopoulos et al.	1	Incomplete sample description

**Table 3:** Summary findings and forest plot of tensile strength (MPa) outcomes in case-control studies incorporating filler particles with MFPSE.

Study or Subgroup	With Filler Particle			Without Filler Particle			Weight	Std. Mean Difference
	Mean	SD	Total	Mean	SD	Total		IV, Random, 95% CI
Haider (2019) 5%PP	4.41	0.21	10	4.89	0.45	10	5.9%	−1.31 [−2.30, −0.32]
Jebur (2018) 0.5%PF	6.11	0.68	10	6.66	0.45	10	6.0%	−0.91 [−1.85, 0.02]
Haider (2019) 3%PP	4.59	0.39	10	4.89	0.45	10	6.0%	−0.68 [−1.59, 0.23]
Alsamaraay (2017) 1%Nylon	5.5	0.81	10	5.74	0.67	10	6.1%	−0.31 [−1.19, 0.57]
Jebur (2018) 0.25%PF	6.81	0.61	10	6.66	0.45	10	6.1%	0.27 [−0.61, 1.15]
Haider (2019) 1%PP	5.01	0.11	10	4.89	0.45	10	6.0%	0.35 [−0.53, 1.24]
Azeez (2018) 0.5% Ag Zn	3.79	0.14	10	3.63	0.25	10	6.0%	0.76 [−0.16, 1.67]
Azeez (2018) 1.5% Ag Zn	3.8	0.13	10	3.63	0.25	10	6.0%	0.82 [−0.10, 1.74]
Alsmael (2018) 1% TiS	7.63	0.71	10	7.01	0.54	10	6.0%	0.94 [0.01, 1.88]
Mohammad (2019) 1% Y_2_O_2_	5.59	0.67	10	4.89	0.74	10	6.0%	0.95 [0.01, 1.89]
Mohammad (2019) 1.5% Y_2_O_2_	5.65	0.66	10	4.89	0.74	10	5.9%	1.04 [0.09, 1.99]
Zayed (2014) 3% SiO_2_	3.62	0.69	21	2.78	0.36	21	6.3%	1.50 [0.81, 2.19]
Alsmael (2018) 0.5% TiS	8.01	0.72	10	7.01	0.54	10	5.8%	1.50 [0.49, 2.52]
Shakir (2018) 0.25%–2%TiO_2_	6.45	0.29	10	5.89	0.28	10	5.7%	1.88 [0.79, 2.97]
Azeez (2018) 1% Ag Zn	4.08	0.13	10	3.63	0.25	10	5.6%	2.16 [1.01, 3.32]
Kareem (2018) 1%ZrS	4.94	0.5	10	3.89	0.11	10	5.3%	2.78 [1.48, 4.08]
Kareem (2018) 0.5%ZrS	4.92	0.35	10	3.89	0.11	10	4.8%	3.80 [2.22, 5.38]
Kareem (2018) 1.5%ZrS	6.72	0.13	10	3.89	0.11	10	0.6%	22.51 [14.68, 30.34]
Subtotal (95% CI)			191			191	100.0%	0.99 [0.36, 1.61]
Heterogeneity: Tau² = 1.48; Chi² = 116.50, df = 17 (*P* < 0.00001); I² = 85%								
Test for overall effect: Z = 3.09 (*P* = 0.002)								

**Table 4:** Subgroup analysis and forest plot of tensile strength (MPa) studies.

Study or Subgroup	With Filler Particle			Without Filler Particle			Weight	Std. Mean Difference
	Mean	SD	Total	Mean	SD	Total		IV, Random, 95% CI
**2.3.1 NANO**								
Alsmael (2018) 1% TiS	7.63	0.71	10	7.01	0.54	10	6.0%	0.94 [0.01, 1.88]
Mohammad (2019) 1% Y_2_O_2_	5.59	0.67	10	4.89	0.74	10	6.0%	0.95 [0.01, 1.89]
Mohammad (2019) 1.5% Y_2_O_2_	5.65	0.66	10	4.89	0.74	10	5.9%	1.04 [0.09, 1.99]
Zayed (2014) 3% SiO_2_	3.62	0.69	21	2.78	0.36	21	6.3%	1.50 [0.81, 2.19]
Alsmael (2018) 0.5% TiS	8.01	0.72	10	7.01	0.54	10	5.8%	1.50 [0.49, 2.52]
Shakir (2018) 0.25%–2%TiO_2_	6.45	0.29	10	5.89	0.28	10	5.7%	1.88 [0.79, 2.97]
Kareem (2018) 1%ZrS	4.94	0.5	10	3.89	0.11	10	5.3%	2.78 [1.48, 4.08]
Kareem (2018) 0.5%ZrS	4.92	0.35	10	3.89	0.11	10	4.8%	3.80 [2.22, 5.38]
Kareem (2018) 1.5%ZrS	6.72	0.13	10	3.89	0.11	10	0.6%	22.51 [14.68, 30.34]
Subtotal (95% CI)			101			101	46.4%	1.96 [1.09, 2.84]
Heterogeneity: Tau² = 1.31; Chi² = 43.43, df = 8 (*P* < 0.00001); I² = 82%								
Test for overall effect: Z = 4.41 (*P* < 0.0001)								
**2.3.2 MICRO**								
Haider (2019) 5%PP	4.41	0.21	10	4.89	0.45	10	5.9%	−1.31 [−2.30, −0.32]
Jebur (2018) 0.5%PF	6.11	0.68	10	6.66	0.45	10	6.0%	−0.91 [−1.85, 0.02]
Haider (2019) 3%PP	4.59	0.39	10	4.89	0.45	10	6.0%	−0.68 [−1.59, 0.23]
Alsamaraay (2017) 1%Nylon	5.5	0.81	10	5.74	0.67	10	6.1%	−0.31 [−1.19, 0.57]
Jebur (2018) 0.25%PF	6.81	0.61	10	6.66	0.45	10	6.1%	0.27 [−0.61, 1.15]
Haider (2019) 1%PP	5.01	0.11	10	4.89	0.45	10	6.0%	0.35 [−0.53, 1.24]
Azeez (2018) 0.5% Ag Zn	3.79	0.14	10	3.63	0.25	10	6.0%	0.76 [−0.16, 1.67]
Azeez (2018) 1.5% Ag Zn	3.8	0.13	10	3.63	0.25	10	6.0%	0.82 [−0.10, 1.74]
Azeez (2018) 1% Ag Zn	4.08	0.13	10	3.63	0.25	10	5.6%	2.16 [1.01, 3.32]
Subtotal (95% CI)			90			90	53.6%	0.11 [−0.53, 0.74]
Heterogeneity: Tau² = 0.71; Chi² = 33.08, df = 8 (*P* < 0.0001); I² = 76%								
Test for overall effect: Z = 0.33 (*P* = 0.74)								
Total (95% CI)			191			191	100.0%	0.99 [0.36, 1.61]
Heterogeneity: Tau² = 1.48; Chi² = 116.50, df = 17 (*P* < 0.00001); I² = 85%								
Test for overall effect: Z = 3.09 (*P* = 0.002)								
Test for subgroup differences: Chi² = 11.40, df = 1 (*P* = 0.0007), I² = 91.2%								

**Table 5:** Summary findings and forest plot of tear strength (N/mm) outcomes in case-control studies incorporating filler particles with MFPSE.

Study or Subgroup	With Filler Particle			Without Filler Particle			Weight	Std. Mean Difference
	Mean	SD	Total	Mean	SD	Total		IV, Random, 95% CI
Alsamaraay (2017) 1%Nylon	20.12	1.89	10	20.99	1.21	10	6.2%	−0.53 [−1.42, 0.37]
Haider (2019) 5%PP	20	0.74	10	20.77	2.4	10	6.2%	−0.42 [−1.30, 0.47]
Jebur (2018) 0.5%PF	25.34	1.45	10	25.97	2.49	10	6.2%	−0.30 [−1.18, 0.59]
Jebur (2018) 0.25%PF	26.81	2.34	10	25.97	2.49	10	6.2%	0.33 [−0.55, 1.22]
Kareem (2018) 1%ZrS	21.66	2.99	10	20.75	0.98	10	6.2%	0.39 [−0.50, 1.28]
Haider (2019) 3%PP	21.95	2.98	10	20.77	2.4	10	6.2%	0.42 [−0.47, 1.31]
Alsmael (2018) 1% TiS	26.48	3.46	10	23.39	2.29	10	6.1%	1.01 [0.07, 1.95]
Kareem (2018) 0.5%ZrS	21.65	0.47	10	20.75	0.98	10	6.1%	1.12 [0.16, 2.08]
Haider (2019) 1%PP	23.48	0.76	10	20.77	2.4	10	6.1%	1.46 [0.45, 2.47]
Alsmael (2018) 0.5% TiS	27.92	2.95	10	23.39	2.29	10	6.1%	1.64 [0.60, 2.69]
Mohammad (2019) 1% Y_2_O_2_	26.89	1.15	10	23.93	1.08	10	5.9%	2.54 [1.30, 3.78]
Kareem (2018) 1.5%ZrS	23.4	0.66	10	20.75	0.98	10	5.8%	3.04 [1.67, 4.41]
Mohammad (2019) 1.5% Y_2_O_2_	27.98	0.67	10	23.93	1.08	10	5.4%	4.32 [2.59, 6.05]
Azeez (2018) 1.5% Ag Zn	19.18	1.09	10	14.81	0.67	10	5.3%	4.63 [2.80, 6.45]
Azeez (2018) 0.5% Ag Zn	19.03	0.85	10	14.81	0.67	10	5.1%	5.28 [3.26, 7.31]
Shakir (2018) 0.25%–2%TiO_2_	27.67	0.47	10	23.61	0.34	10	3.7%	9.48 [6.09, 12.87]
Azeez (2018) 1% Ag Zn	22.57	0.81	10	14.81	0.67	10	3.5%	10.00 [6.43, 13.57]
Zayed (2014) 3% SiO_2_	45.9	1.94	21	19.32	1.9	21	3.9%	13.58 [10.47, 16.69]
Subtotal (95% CI)			191			191	100.0%	2.62 [1.64, 3.59]
Heterogeneity: Tau² = 3.84; Chi² = 208.67, df = 17 (*P* < 0.00001); I² = 92%								
Test for overall effect: Z = 5.23 (*P* < 0.00001)								

**Table 6:** Subgroup analysis and forest plot of tear strength (N/mm) studies.

Study or Subgroup	With Filler Particle			Without Filler Particle			Weight	Std. Mean Difference
	Mean	SD	Total	Mean	SD	Total		IV, Random, 95% CI
**4.2.1 Nano Filler**								
Kareem (2018) 1%ZrS	21.66	2.99	10	20.75	0.98	10	6.2%	0.39 [−0.50, 1.28]
Alsmael (2018) 1% TiS	26.48	3.46	10	23.39	2.29	10	6.1%	1.01 [0.07, 1.95]
Kareem (2018) 0.5%ZrS	21.65	0.47	10	20.75	0.98	10	6.1%	1.12 [0.16, 2.08]
Alsmael (2018) 0.5% TiS	27.92	2.95	10	23.39	2.29	10	6.1%	1.64 [0.60, 2.69]
Mohammad (2019) 1% Y_2_O_2_	26.89	1.15	10	23.93	1.08	10	5.9%	2.54 [1.30, 3.78]
Kareem (2018) 1.5%ZrS	23.4	0.66	10	20.75	0.98	10	5.8%	3.04 [1.67, 4.41]
Mohammad (2019) 1.5% Y_2_O_2_	27.98	0.67	10	23.93	1.08	10	5.4%	4.32 [2.59, 6.05]
Shakir (2018) 0.25%–2%TiO_2_	27.67	0.47	10	23.61	0.34	10	3.7%	9.48 [6.09, 12.87]
Zayed (2014) 3% SiO_2_	45.9	1.94	21	19.32	1.9	21	3.9%	13.58 [10.47, 16.69]
Subtotal (95% CI)			101			101	49.1%	3.53 [2.00, 5.06]
Heterogeneity: Tau² = 4.72; Chi² = 101.76, df = 8 (*P* < 0.00001); I² = 92%								
Test for overall effect: Z = 4.53 (*P* < 0.00001)								
**4.2.2 Micro Filler**								
Alsamaraay (2017) 1%Nylon	20.12	1.89	10	20.99	1.21	10	6.2%	−0.53 [−1.42, 0.37]
Haider (2019) 5%PP	20.0	0.74	10	20.77	2.4	10	6.2%	−0.42 [−1.30, 0.47]
Jebur (2018) 0.5%PF	25.34	1.45	10	25.97	2.49	10	6.2%	−0.30 [−1.18, 0.59]
Jebur (2018) 0.25%PF	26.81	2.34	10	25.97	2.49	10	6.2%	0.33 [−0.55, 1.22]
Haider (2019) 3%PP	21.95	2.98	10	20.77	2.4	10	6.2%	0.42 [−0.47, 1.31]
Haider (2019) 1%PP	23.48	0.76	10	20.77	2.4	10	6.1%	1.46 [0.45, 2.47]
Azeez (2018) 1.5% Ag Zn	19.18	1.09	10	14.81	0.67	10	5.3%	4.63 [2.80, 6.45]
Azeez (2018) 0.5% Ag Zn	19.03	0.85	10	14.81	0.67	10	5.1%	5.28 [3.26, 7.31]
Azeez (2018) 1% Ag Zn	22.57	0.81	10	14.81	0.67	10	3.5%	10.00 [6.43, 13.57]
Subtotal (95% CI)			90			90	50.9%	1.72 [0.50, 2.94]
Heterogeneity: Tau² = 2.92; Chi² = 84.10, df = 8 (*P* < 0.00001); I² = 90%								
Test for overall effect: Z = 2.77 (*P* = 0.006)								
Total (95% CI)			191			191	100.0%	2.62 [1.64, 3.59]
Heterogeneity: Tau² = 3.84; Chi² = 208.67, df = 17 (*P* < 0.00001); I² = 92%								
Test for overall effect: Z = 5.23 (*P* < 0.00001)								
Test for subgroup differences: Chi² = 3.31, df = 1 (*P* = 0.07), I² = 69.8%								

**Table 7:** Summary findings and forest plot of hardness (Shore A) outcomes in case-control studies incorporating filler particles with MFPSE.

Study or Subgroup	With Filler Particle			Without Filler Particle			Weight	Std. Mean Difference
	Mean	SD	Total	Mean	SD	Total		IV, Random, 95% CI
Haider (2019) 1%PP	31.04	1.72	10	30.73	1.72	10	6.2%	0.17 [−0.71, 1.05]
Kareem (2018) 0.5%ZrS	31.25	1.71	10	30.93	1.73	10	6.2%	0.18 [−0.70, 1.06]
Azeez (2018) 0.5% Ag Zn	33.69	0.56	10	33.4	0.61	10	6.2%	0.47 [−0.42, 1.37]
Alsmael (2018) 0.5% TiS	37.11	1.75	10	34.89	2.15	10	6.2%	1.08 [0.13, 2.04]
Azeez (2018) 1% Ag Zn	34.01	0.4	10	33.4	0.61	10	6.2%	1.13 [0.17, 2.09]
Alsmael (2018) 1% TiS	38.89	2.45	10	34.89	2.15	10	6.0%	1.66 [0.61, 2.71]
Jebur (2018) 0.25%PF	43.03	0.43	10	41.33	1.04	10	5.9%	2.05 [0.92, 3.17]
Kareem (2018) 1%ZrS	34.51	1.53	10	30.93	1.73	10	5.9%	2.10 [0.96, 3.24]
Haider (2019) 3%PP	34.31	1.51	10	30.73	1.72	10	5.9%	2.12 [0.98, 3.26]
Kareem (2018) 1.5%ZrS	34.86	1.32	10	30.93	1.73	10	5.8%	2.45 [1.23, 3.66]
Haider (2019) 5%PP	34.67	1.29	10	30.73	1.72	10	5.8%	2.48 [1.26, 3.71]
Azeez (2018) 1.5% Ag Zn	34.89	0.44	10	33.4	0.61	10	5.7%	2.68 [1.41, 3.96]
Alsamaraay (2017) 1%Nylon	44.52	0.79	10	42.15	0.89	10	5.7%	2.70 [1.42, 3.98]
Shakir (2018) 0.25%–2%TiO_2_	29.27	0.53	10	27.21	0.29	10	5.0%	4.62 [2.80, 6.44]
Jebur (2018) 0.5%PF	45.5	0.36	10	41.33	1.04	10	4.7%	5.13 [3.15, 7.11]
Zayed (2014) 3% SiO_2_	29.97	0.38	21	28.09	0.32	21	5.7%	5.25 [3.93, 6.58]
Mohammad (2019) 1% Y_2_O_2_	31.09	0.6	10	27.26	0.4	10	3.8%	7.19 [4.56, 9.83]
Mohammad (2019) 1.5% Y_2_O_2_	32.31	0.54	10	27.26	0.4	10	2.8%	10.18 [6.55, 13.81]
Subtotal (95% CI)			191			191	100.0%	2.58 [1.80, 3.37]
Heterogeneity: Tau² = 2.36; Chi² = 128.24, df = 17 (*P* < 0.00001); I² = 87%								
Test for overall effect: Z = 6.46 (*P* < 0.00001)								

**Table 8:** Subgroup analysis and forest plot of hardness (Shore A) studies.

Study or Subgroup	With Filler Particle			Without Filler Particle			Weight	Std. Mean Difference
	Mean	SD	Total	Mean	SD	Total		IV, Random, 95% CI
**5.1.1 Nano filler**								
Kareem (2018) 0.5%ZrS	31.25	1.71	10	30.93	1.73	10	6.2%	0.18 [−0.70, 1.06]
Alsmael (2018) 0.5% TiS	37.11	1.75	10	34.89	2.15	10	6.2%	1.08 [0.13, 2.04]
Alsmael (2018) 1% TiS	38.89	2.45	10	34.89	2.15	10	6.0%	1.66 [0.61, 2.71]
Kareem (2018) 1%ZrS	34.51	1.53	10	30.93	1.73	10	5.9%	2.10 [0.96, 3.24]
Kareem (2018) 1.5%ZrS	34.86	1.32	10	30.93	1.73	10	5.8%	2.45 [1.23, 3.66]
Shakir (2018) 0.25%–2%TiO_2_	29.27	0.53	10	27.21	0.29	10	5.0%	4.62 [2.80, 6.44]
Zayed (2014) 3% SiO_2_	29.97	0.38	21	28.09	0.32	21	5.7%	5.25 [3.93, 6.58]
Mohammad (2019) 1% Y_2_O_2_	31.09	0.6	10	27.26	0.4	10	3.8%	7.19 [4.56, 9.83]
Mohammad (2019) 1.5% Y_2_O_2_	32.31	0.54	10	27.26	0.4	10	2.8%	10.18 [6.55, 13.81]
Subtotal (95% CI)			101			101	47.5%	3.41 [1.97, 4.84]
Heterogeneity: Tau² = 4.11; Chi² = 86.12, df = 8 (*P* < 0.00001); I² = 91%								
Test for overall effect: Z = 4.64 (*P* < 0.00001)								
**5.1.2 Micro filler**								
Haider (2019) 1%PP	31.04	1.72	10	30.73	1.72	10	6.2%	0.17 [−0.71, 1.05]
Azeez (2018) 0.5% Ag Zn	33.69	0.56	10	33.4	0.61	10	6.2%	0.47 [−0.42, 1.37]
Azeez (2018) 1% Ag Zn	34.01	0.4	10	33.4	0.61	10	6.2%	1.13 [0.17, 2.09]
Jebur (2018) 0.25%PF	43.03	0.43	10	41.33	1.04	10	5.9%	2.05 [0.92, 3.17]
Haider (2019) 3%PP	34.31	1.51	10	30.73	1.72	10	5.9%	2.12 [0.98, 3.26]
Haider (2019) 5%PP	34.67	1.29	10	30.73	1.72	10	5.8%	2.48 [1.26, 3.71]
Azeez (2018) 1.5% Ag Zn	34.89	0.44	10	33.4	0.61	10	5.7%	2.68 [1.41, 3.96]
Alsamaraay (2017) 1%Nylon	44.52	0.79	10	42.15	0.89	10	5.7%	2.70 [1.42, 3.98]
Jebur (2018) 0.5%PF	45.5	0.36	10	41.33	1.04	10	4.7%	5.13 [3.15, 7.11]
Subtotal (95% CI)			90			90	52.5%	1.95 [1.12, 2.78]
Heterogeneity: Tau² = 1.24; Chi² = 38.35, df = 8 (*P* < 0.00001); I² = 79%								
Test for overall effect: Z = 4.61 (*P* < 0.00001)								
Total (95% CI)			191			191	100.0%	2.58 [1.80, 3.37]
Heterogeneity: Tau² = 2.36; Chi² = 128.24, df = 17 (*P* < 0.00001); I² = 87%								
Test for overall effect: Z = 6.46 (*P* < 0.00001)								
Test for subgroup differences: Chi² = 2.97, df = 1 (*P* = 0.08), I² = 66.3%								

**Table 9:** Summary findings and forest plot of elongation at break (%) outcomes in case-control studies incorporating filler particles with MFPSE.

Study or Subgroup	With Filler Particle			Without Filler Particle			Weight	Std. Mean Difference
	Mean	SD	Total	Mean	SD	Total		IV, Random, 95% CI
Azeez (2018) 1.5% Ag Zn	312.9	8.96	10	342.5	9.22	10	9.8%	−3.12 [−4.51, −1.73]
Alsamaraay (2017) 1%Nylon	203.52	23.02	10	270.69	21.43	10	9.9%	−2.89 [−4.22, −1.56]
Azeez (2018) 1% Ag Zn	319.9	6.1	10	342.5	9.22	10	9.9%	−2.77 [−4.07, −1.47]
Azeez (2018) 0.5% Ag Zn	327.9	6.31	10	342.5	9.22	10	10.0%	−1.77 [−2.84, −0.70]
Jebur (2018) 0.25%PF	216.29	10.86	10	213.33	43.33	10	10.1%	0.09 [−0.79, 0.97]
Jebur (2018) 0.5%PF	220.55	11.19	10	213.33	43.33	10	10.1%	0.22 [−0.66, 1.10]
Shakir (2018) 0.25%–2%TiO_2_	1462.71	120.0	10	1431.17	56.9	10	10.1%	0.32 [−0.56, 1.21]
Kareem (2018) 0.5%ZrS	994.74	84.02	10	770.93	59.35	10	9.9%	2.95 [1.60, 4.29]
Kareem (2018) 1.5%ZrS	1424.4	81.72	10	770.93	59.35	10	8.4%	8.76 [5.61, 11.92]
Kareem (2018) 1%ZrS	1229.09	30.63	10	770.93	59.35	10	8.2%	9.29 [5.96, 12.62]
Zayed (2014) 3% SiO_2_	754.8	4.06	21	590.0	3.18	21	3.1%	44.34 [34.36, 54.32]
Mohammad (2019) 1% Y_2_O_2_	569.0	0.5	10	515.9	0.33	10	0.3%	120.05 [78.53, 161.58]
Mohammad (2019) 1.5% Y_2_O_2_	574.0	0.5	10	515.9	0.33	10	0.2%	131.36 [85.92, 176.79]
Total (95% CI)			141			141	100.0%	2.78 [0.66, 4.90]
Heterogeneity: Tau² = 11.37; Chi² = 282.81, df = 12 (*P* < 0.00001); I² = 96%								
Test for overall effect: Z = 2.57 (*P* = 0.01)								

**Table 10:** Subgroup analysis and forest plot of elongation at break (%) studies.

Study or Subgroup	With Filler Particle			Without Filler Particle			Weight	Std. Mean Difference
	Mean	SD	Total	Mean	SD	Total		IV, Random, 95% CI
**1.3.1 NANO**								
Shakir (2018) 0.25%–2%TiO_2_	1462.71	120.0	10	1431.17	56.9	10	10.1%	0.32 [−0.56, 1.21]
Kareem (2018) 0.5%ZrS	994.74	84.02	10	770.93	59.35	10	9.9%	2.95 [1.60, 4.29]
Kareem (2018) 1.5%ZrS	1424.4	81.72	10	770.93	59.35	10	8.4%	8.76 [5.61, 11.92]
Kareem (2018) 1%ZrS	1229.09	30.63	10	770.93	59.35	10	8.2%	9.29 [5.96, 12.62]
Zayed (2014) 3% SiO_2_	754.8	4.06	21	590.0	3.18	21	3.1%	44.34 [34.36, 54.32]
Mohammad (2019) 1% Y_2_O_2_	569.0	0.5	10	515.9	0.33	10	0.3%	120.05 [78.53, 161.58]
Mohammad (2019) 1.5% Y_2_O_2_	574.0	0.5	10	515.9	0.33	10	0.2%	131.36 [85.92, 176.79]
Subtotal (95% CI)			81			81	40.1%	14.65 [8.69, 20.61]
Heterogeneity: Tau² = 43.04; Chi² = 183.03, df = 6 (*P* < 0.00001); I² = 97%								
Test for overall effect: Z = 4.82 (*P* < 0.00001)								
**1.3.2 MICRO**								
Azeez (2018) 1.5% Ag Zn	312.9	8.96	10	342.5	9.22	10	9.8%	−3.12 [−4.51, −1.73]
Alsamaraay (2017) 1%Nylon	203.52	23.02	10	270.69	21.43	10	9.9%	−2.89 [−4.22, −1.56]
Azeez (2018) 1% Ag Zn	319.9	6.1	10	342.5	9.22	10	9.9%	−2.77 [−4.07, −1.47]
Azeez (2018) 0.5% Ag Zn	327.9	6.31	10	342.5	9.22	10	10.0%	−1.77 [−2.84, −0.70]
Jebur (2018) 0.25%PF	216.9	10.86	10	213.33	43.33	10	10.1%	0.11 [−0.77, 0.99]
Jebur (2018) 0.5%PF	220.55	11.19	10	213.33	43.33	10	10.1%	0.22 [−0.66, 1.10]
Subtotal (95% CI)			60			60	59.9%	−1.64 [−2.89, −0.38]
Heterogeneity: Tau² = 2.11; Chi² = 38.76, df = 5 (*P* < 0.00001); I² = 87%								
Test for overall effect: Z = 2.56 (*P* = 0.01)								
Total (95% CI)			141			141	100.0%	2.78 [0.66, 4.90]
Heterogeneity: Tau² = 11.37; Chi² = 282.87, df = 12 (*P* < 0.00001); I² = 96%								
Test for overall effect: Z = 2.57 (*P* = 0.01)								
Test for subgroup differences: Chi² = 27.44, df = 1 (*P* < 0.00001), I² = 96.4%								

## Supplementary Materials

The following are available online at https://www.mdpi.com/2073-4360/12/7/1536/s1, Table S1: Subgroup analysis and forest plot of hardness (Shore A) studies.

## Abbreviations and Acronyms

Ag-Zn	Silver-zinc
BaSO_4_	Barium sulphate
CeO_2_	Cerium oxide
CI	Confidence interval
MFPSE	Maxillofacial prosthetic silicone elastomer
MPa	Megapascal
N/mm	Newton/millimeter
PDMS	Polydimethylsiloxane
PICO	Population, Intervention, Control and Outcome
PMMA	Polymethyl methacrylate
PPP	Pomegranate peels powder
PRISMA	Preferred Reporting Items for Systematic Reviews and Meta-Analyses
RTV	Room Temperature Vulcanizing
SiO_2_	Silica/silicone dioxide
SPDA	Seeds powder of dates Ajwa
TiO_2_	Titanium dioxide
TiSiO_4_	Titanium silicate
UHMWPE	Ultra-high molecular weight polyethylene
Y_2_O_3_	Yttrium oxide
ZnO	Zinc oxide
ZrSiO_4_	Zirconium silicate
