# Supplementary material for: The Influence of Filler Particles on the Mechanical Properties of Maxillofacial Prosthetic Silicone Elastomers: A Systematic Review and Meta-Analysis
Source: Polymers (Basel). 2020 Jul 12;12(7):1536. doi: 10.3390/polym12071536 (PMC7408417; doi:10.3390/polym12071536)
Supplement: Supplementary file 1 [file polymers-12-01536-s001.pdf]

Table S1. Subgroup analysis and forest plot of hardness (Shore A) studies.

Author (year)	Year	Type of Silicone	Filler with %	Tensile Strength (MPa)	Tear Strength (N/mm)	Hardness (Shore A)	Elongation at Break (%)	Author remarks
**Mohammad et al**	**2019**	**VST-50F**	**Y_2O_3 (1%-1.5%)**	**more in 1.5% filler<br><br>5.650 MPa<br>(P > 0.05)**	**More in 1.5% filler<br><br>27.98 N/mm<br>(P<0.05)**	**More in 1.5% filler<br><br>32.31<br>(P<0.05)**	**Increased by 574 for 1.5% concentration (P>0.05)**	**1. Adding Y_2O_3 significantly increase tear and hardness but non-significantly increase in tensile strength.**
Salih et al	2019	VST-50F	Pomegranate Peels Powder (PPP), powder of dates Ajwa (SPDA) (0.0, 0.1%, 0.2%, 0.3%, 0.4%)	More in 0.2% PPP 7.888 MPa,<br><br>0.3% SPDA 7.3 MPa	More in 0.2% PPP 31.1N/mm,<br><br>0.3% SPDA 30.6 N/mm	More in 0.4% PPP 30.5,<br><br>0.3% SPDA 30.00	Not seen	1. Author did not use net silicone, they added PMMA with silicone.<br>2. P value was not mentioned.
Salih et al	2019	VST-50F	Pomegranate Peels Powder (PPP), woven fibers (UHMWPE) (0.0%, 0.5% , 1% UHMWPE fiber, 0.2% PPP)	More in 0.5% UHMWPE chopped fibers - 8.05 MPa<br><br>continuous fibers- 10.4 MPa	More in 0.5% UHMWPE continuous fibers<br><br>48.00 N/mm	More in 1% UHMWPE continuous fibers<br><br>37.00	Not seen	1. Chopped and continuous UHMWPE was used.<br>2. In certain concentrations property increase then decrease.<br>3. Author did not use net silicone. He added PMMA with silicone.<br>4. P value was not mentioned.
**Haider et al**	**2019**	**A-2186**	**Polyester powder (1%,3%,5%)**	**More in 1% filler<br><br>5.010 MPa**	**More in 1% filler<br><br>23.48 N/mm**	**More in 5% filler<br><br>34.6750**	**Not seen**	**1. Polyester showed no significant effect on the hardness of the**

				**(P=0.001)**	**(P=0.002)**	**(P<0.01)**		**silicone, but it was still within the acceptable clinical limit**
**Alsmael et al**	**2018**	**Silicone VST-50**	**Titanium Silicate ($TiSiO_4$) (0.5% -1%)**	**More in 0.5% filler**<br><br>**8.01 MPa**<br>**(P =0.426)**	**More in 0.5% filler**<br><br>**27.92 N/mm**<br>**(P=0.362)**	**More in 1% 38.89**<br>**(P=0.259)**	**Not seen**	**1. Most optimum enhancement of silicone was observed on 0.5% filler.**
Pınar Çevik	2018	A -2000, A -2006	Silane treated silica, fumed silica and TiO_2 (10%)	Not seen	Not seen	More in A-2000 in silane filler<br><br>48.86<br>(P<0.001)	Not seen	1. A-2000 silicone showed maximum hardness values in all study groups.
**Azeez et al**	**2018**	**VST-50**	**Ag-Zn zeolite powder (0.5% - 1.5%)**	**More with 1% filler**<br><br>**4.08 MPa**<br>**(P<0.01)**	**More in 1% filler**<br><br>**22.57 N/mm**<br>**(P<0.01)**	**More in 1.5% filler**<br><br>**34.89**<br>**(P<0.01)**	**More in control group**<br><br>**342.5%**<br>**(P<0.01)**	**1. 1% filler shows change in tear and tensile strength but not change in hardness.**
**Shakir et al**	**2018**	**VST50F, Cosmesil M511**	**nano-TiO_2 (0.25%- 0.2%)**	**More in RTV silicone**<br><br>**6.450 MPa**<br>**(P< 0.01)**	**More in RTV silicone**<br><br>**27.670 N/mm**<br>**(P< 0.01)**	**More in RTV silicone**<br><br>**29.270**<br>**(P< 0.01)**	**Increased by 1462.707 for RTV silicone**<br>**(P<0.01)**	**1. TiO_2 improves tear and tensile strength but increases the hardness is directly proportion to the concentration of the nanofiller**
Salih et al	2018	Silicone rubber	PMMA (5%,10%,15%,20 %)	More in 10% filler	Not seen	More in 10%	More in control group	1. Undefined particle size of PMMA acrylic powder.<br>2. P value was not mentioned.

Al-Hakam J Ibrahim* , Hikmat Jameel Al-Judy	2018	VST-50F	Chitosan powder (1.5%,2.5%,3.5%)	Significant decrease	More in 3.5% filler	Not seen	Not seen	1. Antimicrobial effect is also seen in this paper.<br>2. P value was not mentioned.
**Jebur et al**	**2018**	**A-2186**	**Polyester fibre(0.25%,0.5%)**	**More in 0.25% filler**<br><br>**6.8120 MPa**	**More in 0.25% filler**<br><br>**25.968 N/mm**	**More in 0.5% filler**<br><br>**45.497**	**Increased by 220.551 for 0.5% filler concentrate**	**1. Adding polyester increase tensile and tear strength in certain concentration then decreased but hardness increased proportionally with increased polyester fibers.**<br>**2. P value was not mentioned.**
**Kareem et al**	**2018**	**RTV (Name not mentioned)**	**Zirconium silicate ($ZrSiO_4$) (0.5-1.5%)**	**More in 1.5% filler**<br><br>**6.725 MPa (P<0.01)**	**More in 1.5% filler**<br><br>**23.40 N/mm (P<0.01)**	**More in 1.5% filler**<br><br>**34.86 (P<0.01)**	**Increased by 1424.40 for 1.5% concentration (P<0.01)**	**1. Increases all the properties but highest increase in hardness.**
Pinar Cevik, Oguz Eraslan,	2017	A -2000, A -2006	TiO_2, Silaned silica hydrophobic, Fumed silica hydrophilic (10%)	More in A -2000 silicone with Fumed silica hydrophilic filler<br><br>5.33 MPa (P=0.002)	More in A -2000 silicone with Fumed silica hydrophilic filler<br><br>17.93 N/mm (P= 0.187)	More in A-2000 silicone with Fumed silica hydrophilic filler<br><br>36.64 (P=0,028)	More in control group<br><br>485.33 (P=0.029)	1. A-2000 silicone elastomer revealed maximum mechanical strengths in all study groups.
Mustafa S. Tukmachi	2017	Cosmesil M-511	SiO_2 (0%, 4%, 5%, 6%)	More in 5% filler	More in 5% filler	More in 6% filler	Increased by 728.8 for	1. 5% Nano SiO_2 significantly improves all mechanical properties.

,<br>Mohammed<br>Moudhaffer M. Ali				36.63 MPa<br>(P<0.01)	21.408 N/mm<br>(P<0.01)	36.63<br>(P<0.01)	5% concentration<br>(P<0.01)	2. In one group color change is seen.
**Alsamaraay et al**	**2017**	**A-2186**	**Polyamide-6 (Nylon-6) (1%,3%,5%)**	**Non-significant increase in 1% filler**<br><br>**5.505 MPa**<br>**(P=0.49)**	**Non-significant increase in 1% filler**<br><br>**20.122 N/mm**<br>**(P=1.452)**	**Increased in 1% filler**<br><br>**44.52**<br>**(P<0.01)**	**More in control group**<br>**270.69**<br>**(P<0.01)**	**1. PA-6 micro fillers prevent rapid degradation of mechanical properties of silicone under aging condition.**<br>**2. Variation in the mechanical properties of silicone value is directly proportional to the dose and duration of the radiation.**
Kalamarz et al	2016	polydimethylsiloxane, Methylhydrosiloxane	hydrophobic fumed silica, silanamine (10%, 15%)	More with 15% hydrophobic fumed silica<br><br>(P<0.01)	Not seen	More with 15% hydrophobic fumed silica<br><br>(P<0.01)	Not seen	1. Change in properties was seen after 24 hours, after 7 days and after 28 days.<br>2. Particular value not mentioned only bar chart given
Nobrega et al	2016	MDX4-4210	ZnO, barium sulphate ($BaSO_4$), TiO_2 (1-2%)	Not seen	More in 1% barium sulphate<br><br>3.11 N/mm<br>(P=0.01)	More in 2% ZnO<br><br>29.33<br>(P<0.001)	Not seen	1. Oil paint was used in one group.<br>2. Only hardness was seen with and without incorporated oil paint.
Liu et al	2015	MDX4-4210	Expancel , SiO_2 (5%,10%,15% by volume)	More in 10% SiO_2	More in control group	More in SiO_2 15%	Not seen	1. SiO_2 is better than expancel.<br>2. specific value was not mentioned.

Wang et al	2014	MDX4-4210	TiO_2 (2%,4%,6%)	More in 6% filler<br>3.29 MPa<br>(P < 0.05)	More in 2% filler<br>2.58 N/mm<br>(P < 0.05)	More in 6% filler<br>31.97<br>(P < 0.05)	Increased by<br>254.28 for 2% concentration<br>(P<0.05)	1. Author also seen UV aging<br>2. Inadequate description of sample.
**Zayed et al**	**2014**	**A-2186**	**SiO_2 (0.5%, 1%,1.5%,2%,2.5 %,3%)**	**More in 3% filler**<br>**3.62 MPa**<br>**(P<0.001)**	**More in 3% filler**<br>**45.90 N/mm**<br>**(P<0.001)**	**More in 3% filler**<br>**29.97**	**Increased by**<br>**754.8 for 1.5% concentration**	**1. The correct filler concentration improves mechanical properties.**<br>**2. P value of hardness and elongation at break is not mentioned.**
Liu et al	2013	MDX4-4210	Expancel (0%,5%,15%,30% by volume)	More in 5% filler<br>(P<0.05)	Decrease<br>(P<0.05)	Decrease<br>(P<0.05)	More in 5% filler<br>(P<0.05)	1. Specific values were not mentioned, only graphical representation given.
Han et al	2008	A-2186	TiO_2, ZnO, CeO_2 (0.5% -3%)	More in 2% ZnO 1.7 MPa,<br>More in 2% TiO_2 1.7 MPa<br>(P<0.05)	More in 2% TiO_2<br>2.4 N/mm<br>(P<0.001)	More in 3% CeO_2<br>3.9<br>(P<0.001)	Increased by 59 for<br>2% TiO_2<br>(P<0.001)	1. Incorporating Nano-oxides with silicone elastomer to improve mechanical properties is concentration specific, not dependent on choice of Nano oxide: TiO_2, ZnO, or CeO_2
Gunay et al	2008	A-2186	Tulle % not mentioned	Increased<br>(P<0.05)	Increased<br>(P<0.05)	Not seen	More in control group<br>371<br>(P<0.05)	1. tulle increased resistance against tearing and rupture at the edges without causing any esthetic deformation

Aziz et al	2003	Cosmesil (HC), Cosmesil (Standard)	silica R104, R106, R202, R972, R974, R812s (0%, 5%, 10%, 15%, 20%, 25%)	More in silica filler 20% R812s with 30% DMS<br><br>4.137 MPa (P>0.05)	More in silica filler R812s 20-25%<br><br>7.41 N/mm (P<0.001)	More in silica 20% R812s with 50% DMS<br><br>68.15 (P<0.001)	More in control group<br><br>475.9 (P<0.001)	1. Author used various type of silica filler.<br>2. They used (0%, 5%, 10%, 15%, 20%, 25%) only for tear strength but only 20% R812s for tensile and hardness.
Andreopoulos et al	1998	A poly (dimethyl siloxane)	Silica (up to 50%) by volume	More in 35% silica	More in 50% silica	Not seen	More in 35% silica	1. Specific measurement of properties was not mentioned.<br>2. tensile strength increased up to 35% but tear strength increased with the increase of filler<br>3. Inadequate description of sample size.
Andreopoulos et al	1994	A poly (dimethyl siloxane) rubber	Silica (30%,35%,40%,45%,50%,55%) by volume	More in 55% silica	More in 55% silica	More in 55% silica	More in 35% silica	1. Specific measurement of properties was not mentioned.<br>2. They also used aramid, UHMPE and glass as a reinforcement.<br>3. Silica loading up to 35% increases mechanical strength, which is then decreased.<br>4. Inadequate description of sample size.
